# Properties and performance of a questionnaire assessing COVID-19 vaccine knowledge and attitudes in Brazilian pregnant women

**DOI:** 10.1186/s12889-025-25102-z

**Published:** 2025-11-24

**Authors:** Bruna Maudonnet, Carla Silveira, Jose G. Cecatti, Maria L. Costa, Ricardo P. Tedesco, Jacinta P. Matias, Mateus Tosetto, Ana L. Arthur, Maria Eduarda De Sousa, Rupali J. Limaye, Jessica L. Schue, Emily Miller, Prachi Singh, Berhaun Fesshaye, Ruth A. Karron, Sami L. Gottlieb, Vanessa Brizuela, Ibukun O. Abejirinde, Grace Belayneh, Renato T. Souza

**Affiliations:** 1 Department of Obstetrics and Gynecology, University of Campinas, 101 Alexander Fleming St, Campinas, SP Brazil; 2 Jundiaí School of Medicine - HU/FMJ, Jundiaí, SP Brazil; 3 Department of International Health, Bloomberg School of Public Health, Johns Hopkins University, Baltimore, USA; 4 Center for Immunization Research, Department of International Health, Johns Hopkins Bloomberg School of Public Health, Baltimore, MD USA; 5 Development and Research Training in Human Reproduction (HRP), Department of Sexual and Reproductive Health and Research, UNDP-UNFPA-UNICEF-WHO-World Bank Special Programme of Research, World Health Organization, Geneva, Switzerland

**Keywords:** Psychometric properties, Internal validation, COVID-19 vaccine, Pregnant women

## Abstract

**Objectives:**

This paper aims to evaluate the performance of a questionnaire about pregnant Brazilian women’s attitudes towards and knowledge of COVID-19 vaccines. The findings highlight the importance of tailored psychometric instruments to accurately capture the unique concerns and cultural context of this population. Such tools are essential for informing policymakers in developing targeted strategies and campaigns to increase COVID-19 vaccine uptake during pregnancy.

**Methods:**

A cross-sectional survey of COVID-19 vaccine knowledge, attitudes, and practices took place among pregnant women in two hospitals in southeast Brazil, between August and December 2023. We used a structured approach to evaluate 27 survey questions related to attitudes toward COVID-19 vaccines and sources of knowledge, answered on a four-point Likert scale. We used Cronbach’s alpha, in addition to factorial analysis, to measure sampling adequacy (Kaiser-Meyer-Olkin, KMO). Pearson’s correlation coefficient was used to evaluate the content and correlations between both groups of the instrument among women who had been vaccinated in pregnancy and those who had vaccine hesitancy.

**Results:**

The 27-item questionnaire demonstrated high reliability (Cronbach`s alpha = 0.912), with potential redundancy identified in seven questions. Removing these questions increased the Cronbach`s alpha to 0.921 for the 20-item questionnaire. The 20-item scale showed distinct score distributions between vaccine-hesitant and vaccinated pregnant women, with significant differences (*p* < 0.001). Confirmatory factor analysis of the 20-item scale yielded a high KM0 value (0.908) and was significant using Bartlett`s test (*p* < 0.001), grouping questions into five factors related to vaccine safety, impact on the baby, influence of social circles, trust in information sources and recommendations by authorities. The ROC curve analysis showed the 27-item scale and 20-item scale had an AUC of 0.714 and 0.731, respectively.

**Conclusion:**

The 20-item instrument demonstrated strong reliability and internal consistency, making it a valuable tool for assessing COVID-19 vaccine attitudes among pregnant women in Brazil. Its concise format may enable easier administration without compromising accuracy. Further validation could enhance its role in informing vaccination strategies and improving maternal and neonatal outcomes.

**Supplementary Information:**

The online version contains supplementary material available at 10.1186/s12889-025-25102-z.

## Introduction

Vaccination against SARS-CoV-2 has been instrumental in controlling the severity and spread of COVID-19, significantly reducing mortality rates [1]. Although Brazil is known for its robust immunization policies, the rise of anti-vaccine movements—driven by political factors, denialist movements, misinformation, and concerns over the rapid development of vaccines—has posed challenges in recent years [2]. Despite higher risks of complications due to illness in pregnancy [3], pregnant women often hesitate to vaccinate, even with evidence confirming safety and effectiveness of available vaccines [4–10]. Some of the reasons for hesitancy are concerns around impact of drugs on the fetus and lack of strong recommendations from healthcare providers driven by inadequate training, ambiguous guidelines, and systemic constraints [11, 12].

Vaccine acceptance or hesitancy are complex constructs that differ across context [13, 14]. Factors associated with acceptance or hesitancy occur at multiple levels: individual (e.g., perception of benefits and safety), interpersonal (e.g., influence of family, friends, and religion), health system (e.g., healthcare provider recommendations), and political (e.g., government policies) [15, 16]. A multi-level approach provides a broader understanding of vaccine uptake across diverse contexts. In Kenya, vaccine hesitancy was influenced by individual fears, including infertility and mortality, compounded by limited vaccine accessibility within the healthcare system [15]. In contrast, in Bangladesh, interpersonal influences, particularly the role of a partner, had a significant impact on vaccination decisions [17]. This study aims to investigate the factors contributing to vaccine hesitancy among pregnant women in Brazil [17].

During the study period, Brazil’s sociopolitical landscape was marked by significant polarization and anti-science rhetoric, which undermined public trust in health authorities and contributed to vaccine hesitancy [18]. The federal government’s dismissal of COVID-19 severity, promotion of unproven treatments, and inconsistent public health messaging created widespread confusion. Additionally, the decentralized approach to pandemic management led to inconsistent policies across states and municipalities, further eroding public confidence [18]. Misinformation and conspiracy theories, amplified by social media, exacerbated fears about vaccine safety and efficacy, particularly in communities with limited access to reliable information. These factors, combined with Brazil’s deep socioeconomic inequalities, created a complex environment that influenced vaccine hesitancy and ultimately shaped the country’s COVID-19 outcomes [18].

The World Health Organization’s Strategic Advisory Group of Experts (SAGE) developed the 5 C model to further address vaccine hesitancy. This model encompasses complacency (low perceived risk of the disease), confidence (trust in the vaccine and healthcare system), collective responsibility (concern for community safety), calculation (seeking information), and convenience (access and structural barriers) [19–21]. While 5 C-based tools assess vaccine decisions, adapting these to cultural and contextual specificities is crucial for accuracy [22, 23]. It is also essential to validate the psychometric properties of these instruments to ensure they accurately measure the intended outcomes [24].

Although various questionnaires exist to assess general vaccination attitudes, there is a lack of instruments specifically tailored to measure COVID-19 vaccine hesitancy in pregnant women, who have unique concerns related to pregnancy and fetal health. This study aims to evaluate the psychometric properties of a survey tool about pregnant Brazilian women`s attitudes towards and knowledge of COVID-19 vaccines. The findings will offer valuable insights for policymakers to design more effective vaccine campaigns and policies aimed at improving vaccine uptake during pregnancy by comprehensively assessing the multifaceted factors that influence the decision-making process for vaccination.

## Methods

We used factor analysis to assess the performance of a tool used to collect data on attitudes and knowledge on COVID-19 vaccination among pregnant women in Brazil. This tool was used as part of a larger, cross-sectional study implemented in four countries in collaboration with Johns Hopkins Bloomberg School of Public Health (JHBSPH), and support from the World Health Organization (WHO). Detailed information of the protocol of this research initiative has been published elsewhere [25].The participating health facilities in Brazil encompass specialized and general obstetric antenatal care units affiliated with two maternity hospitals in São Paulo: CAISM/Unicamp Hospital in Campinas and University Hospital of Jundiaí. Both facilities are public hospitals providing care for pregnant women from urban and semi-rural areas, covered by the National Health System (SUS). Data was collected between August and December of 2023.

Participants were recruited from populations who received high-risk antenatal care and specialized care at CAISM/Unicamp and University Hospital of Jundiaí, as well as from two basic healthcare units at Jundiaí serving the general population. Sampling occurred in the waiting area of the clinics and adopted an every “n” approach. The sample size was calculated based on the ability to distinguish the difference in proportion of respondents with a specific attitude comparing two groups, assuming a 50% prevalence of the attitude for maximum variability, with 80% power, a 5% margin of error, and 95% confidence level. Considering these assumptions, we identified the need to include at least 385 participants, which was rounded to 400 to guarantee a sufficient sample size. The inclusion criteria for pregnant women in this study required meeting all of the following conditions: possessing knowledge about COVID-19 vaccination, being 18 years of age or older (or legally emancipated), receiving antenatal care at the selected sites, and having the capacity to provide informed consent. There were no exclusion criteria. Due to ethical restrictions, we were unable to register information regarding the reasons for non-participation.

All participants gave informed consent for inclusion before participating in the study. The study was conducted in accordance with the Declaration of Helsinki, and the protocol was approved by the Ethics Committee of UNICAMP (CAAE 63968222.1.1001.5404) and Jundiaí School of Medicine (CAAE 63968222.1.2001.5412), the PAHO/ERC (Approval letter number 0633.01), the WHO/HRP RP2 assessment (ID A66042-0791), and the Johns Hopkins Bloomberg School of Public Health IRB (approval letter 20864).

A quantitative tool including a maximum of 76 quantitative questions including attitudes towards and knowledge about COVID-19 vaccines was collaboratively developed by all partners involved in the multicenter study. This instrument was developed based on multiple established models, as the constructs of interest were present across these models. Models used include de the 5Cs, Behavior and Social Drivers of Vaccination (BeSD), the Health Belief Model (HBM), and the Socioecological model [16, 20, 26–28]. The survey questionnaire was developed in English; the local team of Brazilian researchers translated it into Brazilian Portuguese. A local team comprised of multidisciplinary experts (general doctors, obstetricians, obstetric nurse, sociologist, literary scholar, and social communication and public health specialist) revised the Portuguese version; most of the team has more than 20 years of experience in developing and applying survey instruments. A pilot evaluation of the instrument was conducted with a sample of individuals (including professors, post-graduate students, and undergraduate students, and four pregnant women with similar eligibility criteria of the study).

The entire instrument is available elsewhere [29]. The first part of the questionnaire gathered sociodemographic information, including age (< 18y or emancipated, 18-34y, 25-34y and >34y), gender, marital status (currently with and without partner), trimester of pregnancy at inclusion (first trimester, ≤ 14 weeks; second trimester, 15–27 weeks; and third, ≥ 28 weeks), number of children under 18 (categorized into no children < 18y or with children < 18y), educational attainment (primary, secondary and college or more), and ethnicity (self-reported as white, pardo or black or other). For this analysis, COVID-19 vaccination status, including whether they received COVID-19 vaccine, number of doses, timing (before and/or during pregnancy), and perceptions of COVID-19 vaccinations were used. COVID-19 perceptions include 19 questions on attitudes and 8 on knowledge, each scored using a four-point Likert scale (strongly agree, agree, disagree, strongly disagree). The tool was implemented among study participants; data were collected on paper and entered into a REDCap database by two independent researchers. In the case of any data inconsistencies, a third researcher reconciled the two entries and corrected any errors.

For the current analysis, we carried out a factor analysis on 19 questions concerning attitudes towards the COVID-19 vaccine and 8 questions of COVID-19 vaccine knowledge and information sources. These 27 questions were answered with a four-point Likert scale, and arbitrarily encoded as: 1 - strongly disagree, 2 - disagree, 3 - agree, 4 - strongly agree. Five questions were written with an inversed correlation; therefore, they were reverse scored for adequacy. We then created a scale based on the sum of the 27-item questionnaire ranging from 27, when the woman strongly disagreed with statements in favor of vaccination to 108, when she strongly agreed. The factor analysis assessed the performance of the set of questions, specifically examining how well the questions predicted the decision to receive the vaccine during pregnancy. We also aimed to identify groupings (factors) of interrelated variables. Afterwards, a reliability analysis of the 27 questions was conducted. To determine whether any item should be excluded from the scale, we identified where internal consistency increased based on Cronbach’s alpha when variables were removed. This evaluation was repeated until there was no further improvement to internal consistency when additional items were removed. Finally, factor analysis and the performance of the final instrument were tested again. In conducting factor analysis, factor loadings > 0.4 were considered significant for item retention. Then, we selected items based on the highest factor loading to ensure that each item is appropriately associated with its respective factor. High factor loadings indicate a strong correlation between the item and the underlying construct represented by the factor, while lower factor loadings suggest weaker associations with other factors. By prioritizing items with the highest loadings, we aimed to enhance the construct validity of the factor analysis, thereby confirming that each selected item is distinctly aligned with the intended factor rather than being influenced by other constructs. This approach minimizes the risk of cross-loading, ensuring that the items contribute meaningfully to the interpretation of the factors derived from the analysis.

The factor analysis began with a measurement of sampling adequacy using the Kaiser-Meyer-Olkin (KMO) test, where values between 0.8 and 0.9 are considered optimal and values above 0.9 are considered excellent (17). The method used for factor extraction was the Varimax method with Kaiser normalization [30]. To determine the number of factors to retain, we employed a combined approach, utilizing both the Kaiser criterion (eigenvalues greater than 1) and an examination of the scree plot to identify the point of inflection, where a natural bend occurs in the eigenvalue distribution; we analyzed the rate of change (slope) across the distribution, specifically looking for points of significant variation [30–32]. The Pearson correlation coefficient was used to measure the correlations between all groupings of the question set. Values above a set cut-off point of 0.7 were considered good correlations. A Bartlett test was used to determine whether the correlation matrix is significantly different from the identity matrix. Values of *p* < 0.05 were considered statistically significant.

**Fig. 1:**
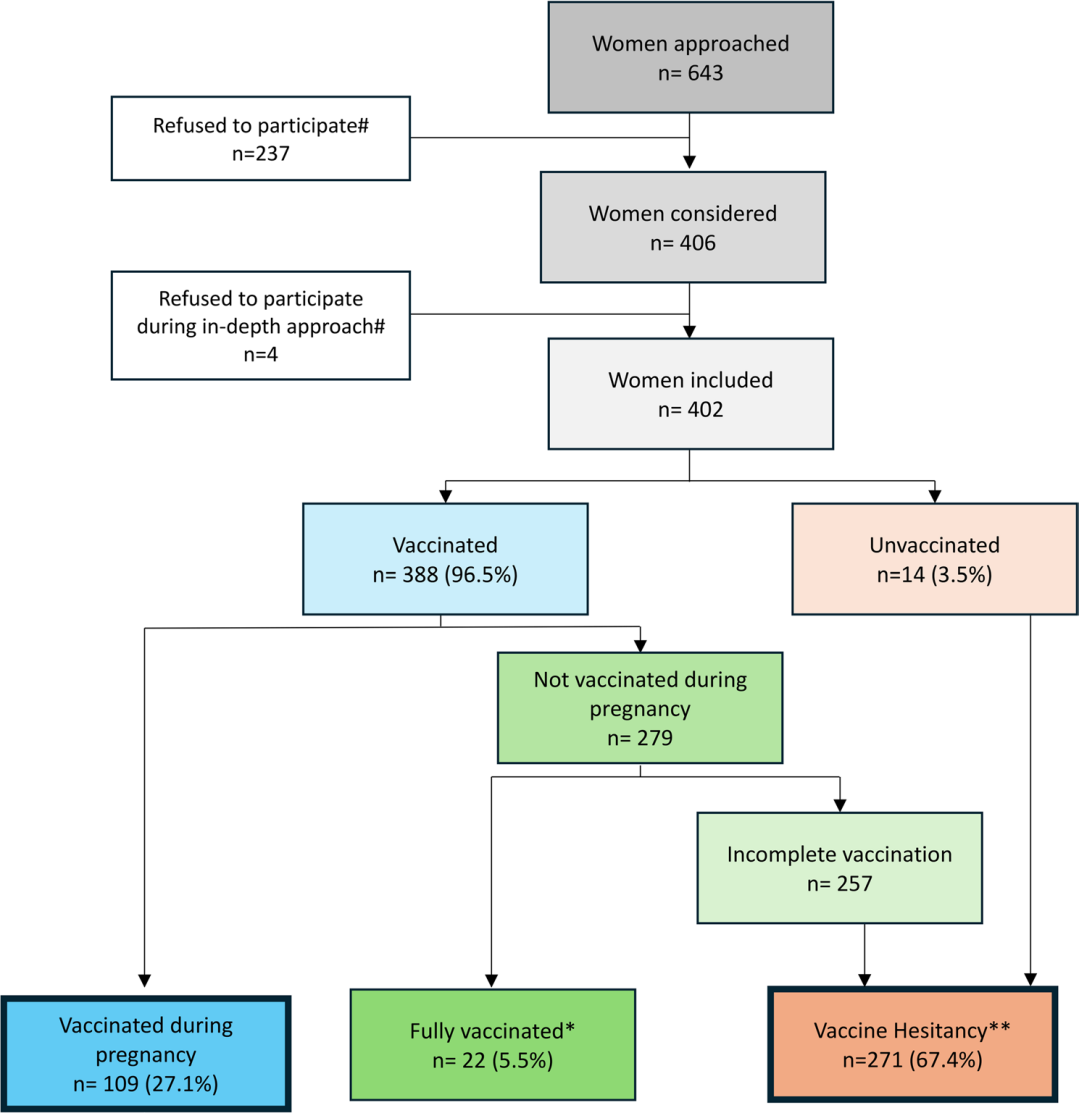
Flowchart of the study population *Fully vaccinated: women with at least three doses of COVID-19 vaccine, including a booster of Pfizer bivalent **Vaccine Hesitancy: women with less than 3 doses of vaccine or without the booster of Pfizer bivalent and who did not receive the vaccine during pregnancy #Reasons for refusal were not available

For our factor analysis, vaccine hesitancy during pregnancy was defined as the outcome variable; we defined hesitancy as pregnant women who were not vaccinated during pregnancy despite an incomplete vaccination schedule according to Brazilian recommendations (Fig. 1). Incomplete vaccination was defined as women who received fewer than three doses of the COVID-19 vaccine or did not receive the booster with bivalent Pfizer [33].

The performance of the factor analysis was assessed with a receiver operating characteristic (ROC) curve and associated values of sensitivity, specificity, accuracy, and positive and negative predictive values. A positive test was defined as the identification of women who were not vaccinated during pregnancy (hesitancy group, as defined above); different cut-offs were assessed according to the coordinates of the ROC curve and the values below a certain cut-off were considered a positive test. Cut-off points were chosen to illustrate their performance across different sensitivity and specificity levels. After examining the coordinates of the resulting ROC curve of the 20-item scale, cut-offs were strategically chosen to capture the turning points at which there is a trade-off between high sensitivity and low specificity, so we can demonstrate the points with most balanced performance.

We used the SPSS version 20.0 software for this analysis (IBM Corp. Released 2011. IBM SPSS Statistics for Windows, Version 20.0. Armonk, NY: IBM Corp.).

## Results

Of the 643 pregnant women approached, 402 (62.5%) agreed to participate. The distribution across gestational trimesters was as follows: 134 (33.3%) in the first trimester, 135 (33.6%) in the second trimester, and 133 (33.1%) in the third trimester (see Table S1, Supplementary Material, and Fig. 1for detailed participant flow). In relation to COVID-19 vaccination status, 14 were never vaccinated and 388 were vaccinated, 109 were vaccinated during pregnancy and 279 were not vaccinated during pregnancy (of these, 22 were fully vaccinated and 257 were incompletely vaccinated (Fig. 1). We did not encounter any missing information in this analysis, either for sociodemographic and pregnancy information, or for other responses to the questionnaire.

The 27-item scale exhibited high internal consistency (Cronbach’s alpha = 0.912) (Table 1). The removal of seven redundant items increased the alpha to 0.921; however, the marginal increase suggests that the primary advantage of the shortened 20-item version is reduced participant burden and improved applicability in field settings. There was no evidence that deleting other questions would increase internal consistency. The questions removed were related to (1) perception of the risks of COVID-19 disease in pregnant women, as the concept that COVID-19 is dangerous for pregnant women seems widespread (three questions); (2) confidence in receiving the vaccine during pregnancy in relation to her health and the fetus (two questions); (3) concern that the vaccine could negatively affect fertility (one question); and (4) trust in media information about vaccination (one question). Table S2 demonstrates the Pearson’s correlation coefficient between questions of the 20-item questionnaire (Supplementary Material).

**Table 1.**
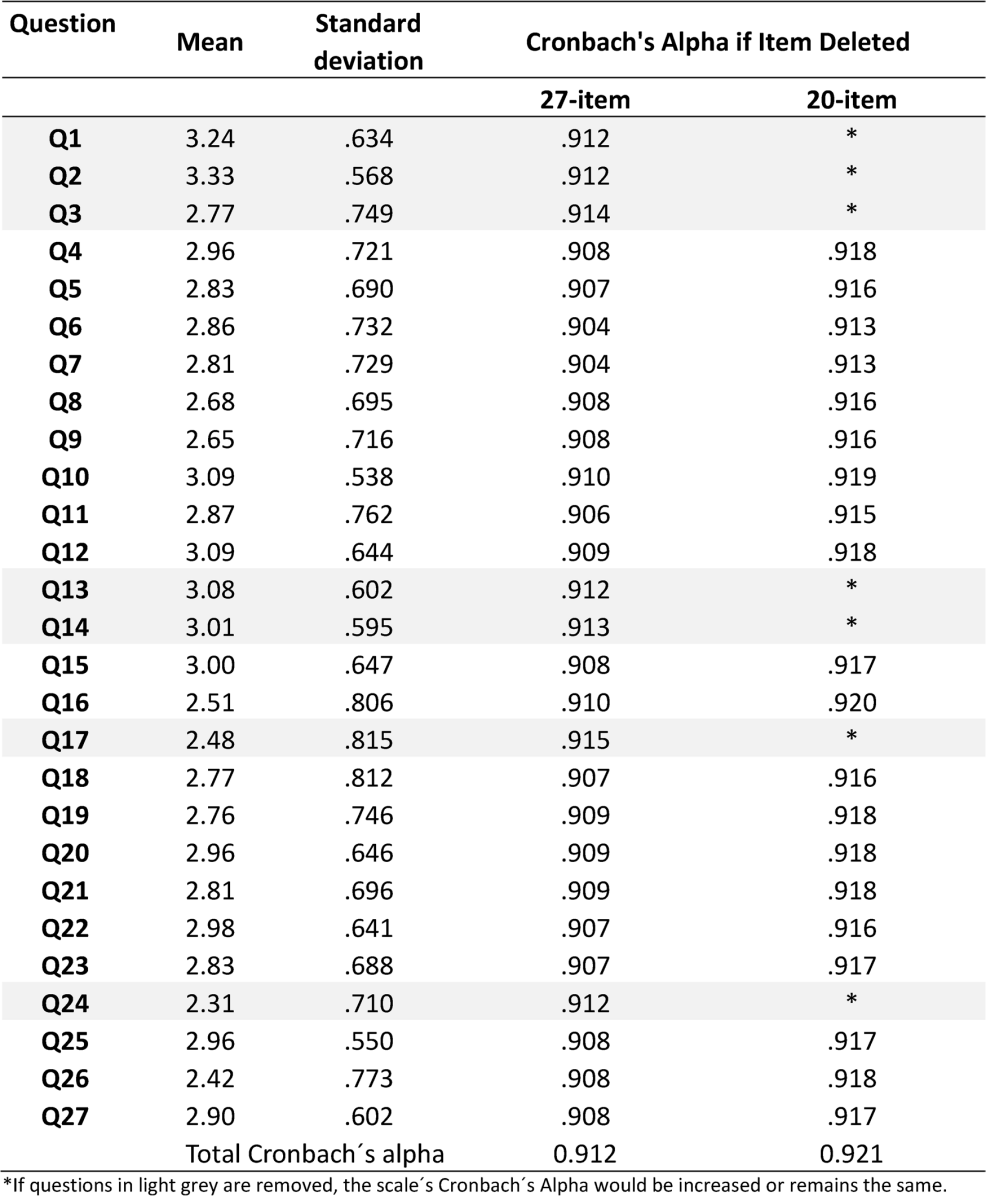
Reliability of the 27-item questionnaire (Cronbach´s Alpha of 0.912; N=402)

*If questions in light grey are removed, the scale´s Cronbach´s Alpha would be increased or remains the same

**Fig. 2:**
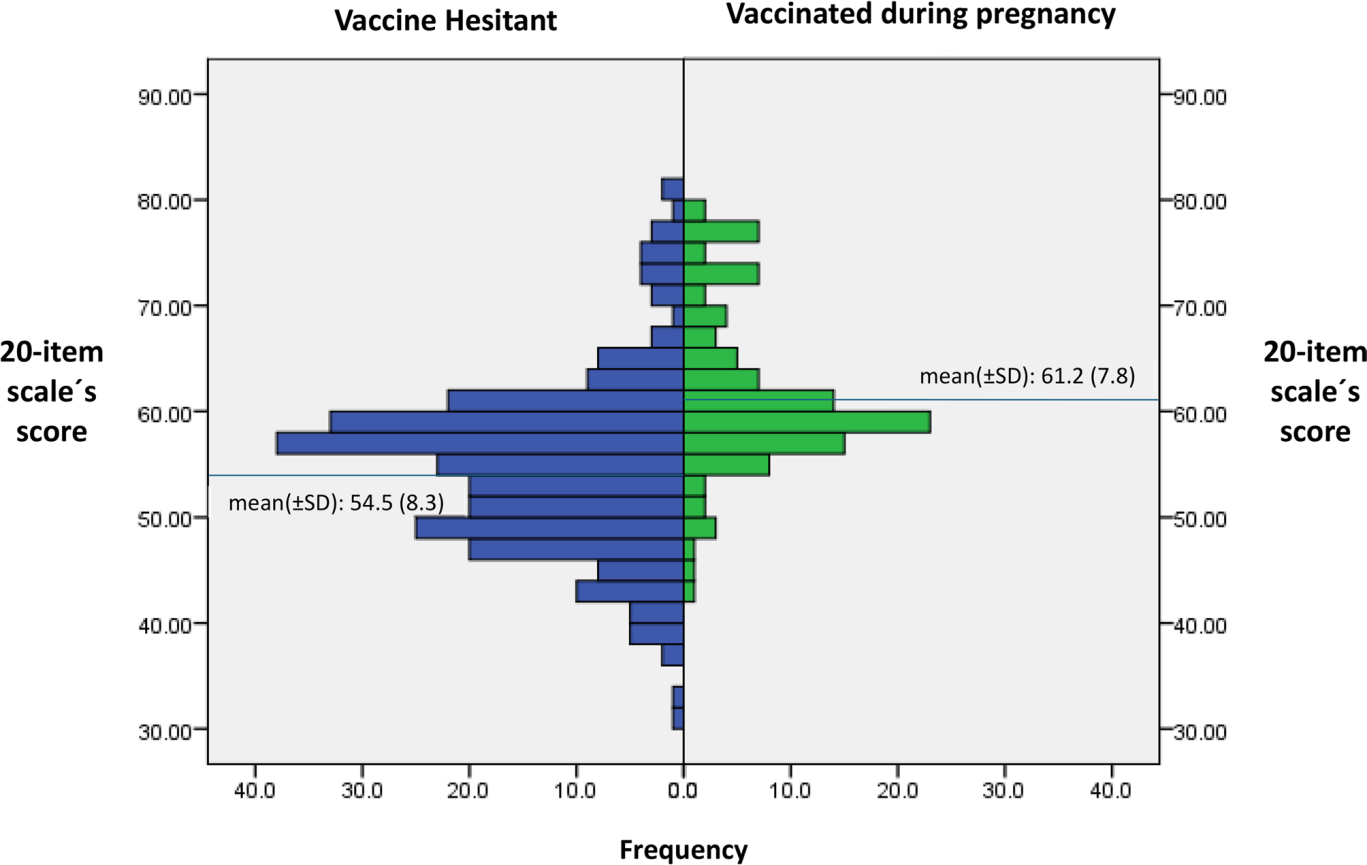
Distribution of the 20-item scale´s score according to vaccination status

The mean (± SD) and minimum-maximum score values of the 27-item and 20-item scales were 76.9 (± 10.2), 48.0—105.0 and 56.7 (± 8.7), 31.0–80.0, respectively. Figure 2 shows the distribution of the 20-item scale according to vaccination status (vaccine hesitant and vaccinated during pregnancy). Pregnant women who received the COVID-19 vaccine during pregnancy had significantly higher mean scores on the 20-item scale (61.2 ± 7.8) compared to the vaccine-hesitant group (54.5 ± 8.3), suggesting greater acceptance and positive perception of vaccination among the vaccinated group (*p* < 0.001).

Confirmatory factor analysis of the 20-item scale yielded a high KMO value (0.908) and was significant using Bartlett’s test (*p* < 0.001) (Table 2), grouping questions into five factors related to vaccine safety, impact on the baby, influence of social circles, trust in information sources, and recommendations by authorities (Table 2). Figure S1 presents the scree plot, displaying the graphical representation of eigenvalues against the corresponding principal components, where a change in slope can be observed between the fifth and sixth factors. The variance explained by each factor is relatively close in value, suggesting a more balanced contribution across the factors; the variance explained by each factor ranged from 8.5% to 13.8%, which indicates that no single factor dominates the variance explanation. The cumulative variance was deemed acceptable, as the extracted factors explained more than half of the variability in the data (54.012%). Questions in factor 1 addressed beliefs about the effectiveness and safety of the vaccine for both the pregnant individual and their baby (perceived benefits and safety). Question in factor 2 covered fears about vaccine ingredients, fertility, and broader skepticism about vaccination (concerns and misconceptions). Factor 3 groups questions that reflect the role of social networks, family, and community support in shaping vaccination decisions (social and community influences). Question from factor 4 addressed the importance of trust in healthcare providers, scientists, and the adequacy of information. Finally, question in factor 5 focuses on the influence of government, politicians, and community leaders on vaccination decisions.

**Table 2:** Confirmatory factor analysis of the 20-item questionnaire (*N* = 402)

		Factors				
	Questions	1	2	3	4	5
**Q4**	Getting the COVID-19 vaccine will reduce my risk of getting COVID-19 during my pregnancy.	**0.550**	− 0.045	0.316	0.199	0.258
**Q5**	Getting the COVID-19 vaccine while I am pregnant will reduce my baby’s risk of getting COVID-19.	**0.550**	0.502	0.180	0.154	0.113
**Q6**	I am confident that getting the COVID-19 vaccine during my pregnancy is safe for me.	**0.549**	0.081	0.333	0.220	0.174
**Q7**	I am confident that getting the COVID-19 vaccine during my pregnancy is safe for my baby.	**0.543**	0.311	0.124	0.038	0.071
**Q8**	I worry that the ingredients in the COVID-19 vaccine given to me during pregnancy are not safe for me.	**0.505**	0.312	0.041	0.198	0.273
**Q9**	I worry that the ingredients in the COVID-19 vaccine given to me during pregnancy are not safe for my baby.	0.109	**0.741**	0.210	0.226	0.181
**Q10**	I worry that the ingredients in the COVID-19 vaccine may negatively impact my fertility.	0.180	**0.731**	0.132	0.261	0.128
**Q11**	I do not want to put the COVID-19 vaccine into my body when I am pregnant because I think it is unnatural.	0.471	**0.493**	0.427	0.238	0.071
**Q12**	Vaccines improve your body’s ability to fight off diseases; this is known as immunity. I believe it is better for my body to develop immunity by getting sick than by getting the COVID-19 vaccine.	0.416	**0.445**	− 0.003	− 0.050	0.150
**Q15**	If I wanted to get the COVID-19 vaccine or a booster dose, and it was available in my community for pregnant people, I am confident I could get the vaccine.	0.214	0.089	**0.661**	0.021	0.317
**Q16**	The majority of my friends would encourage me to get the COVID-19 vaccine during my pregnancy.	0.040	0.116	**0.583**	0.209	0.155
**Q18**	My family would encourage me to get the COVID-19 vaccine during my pregnancy.	0.433	0.447	**0.475**	0.245	0.109
**Q19**	The majority of my pregnant friends and family would get or have gotten the COVID-19 vaccine while they are pregnant.	0.354	0.281	**0.444**	0.166	0.090
**Q20**	I have/had most of the important information I need/needed to make a decision about the COVID-19 vaccine during pregnancy.	0.411	0.168	**0.425**	0.276	0.129
**Q21**	I know enough about the safety of the COVID-19 vaccine to make/have made a decision about getting the vaccine for myself while pregnant.	0.237	0.228	0.110	**0.681**	0.128
**Q22**	I trust the information that I have received from my health care provider about use of the COVID-19 vaccine during pregnancy.	0.041	0.107	0.172	**0.605**	0.086
**Q23**	I trust the information provided by scientists about vaccines during pregnancy.	0.279	0.192	0.151	**0.487**	0.352
**Q25**	The government recommends pregnant people receive the COVID-19 vaccine while pregnant.	0.151	0.100	0.322	0.026	**0.707**
**Q26**	I trust my government and politicians’ recommendations on COVID-19 vaccination for pregnant women.	0.115	0.170	0.194	0.291	**0.606**
**Q27**	Leaders in my community recommend that pregnant people receive the COVID-19 vaccine while pregnant.	0.414	0.221	0.069	0.207	**0.517**
**% Variance** ^a^		13.844	12.479	10.480	8.666	8.544
**% Cumulative variance** ^a^		13.844	26.323	36.803	45.468	54.012

Overall Kaiser-Meyer-Olkin = 0.908; Bartlett’s test of sphericity < 0.001

Values in bold for the same column represent the questions comprising the same factor

^a^Rotation Sums of Squared Loadings

**Fig. 3:**
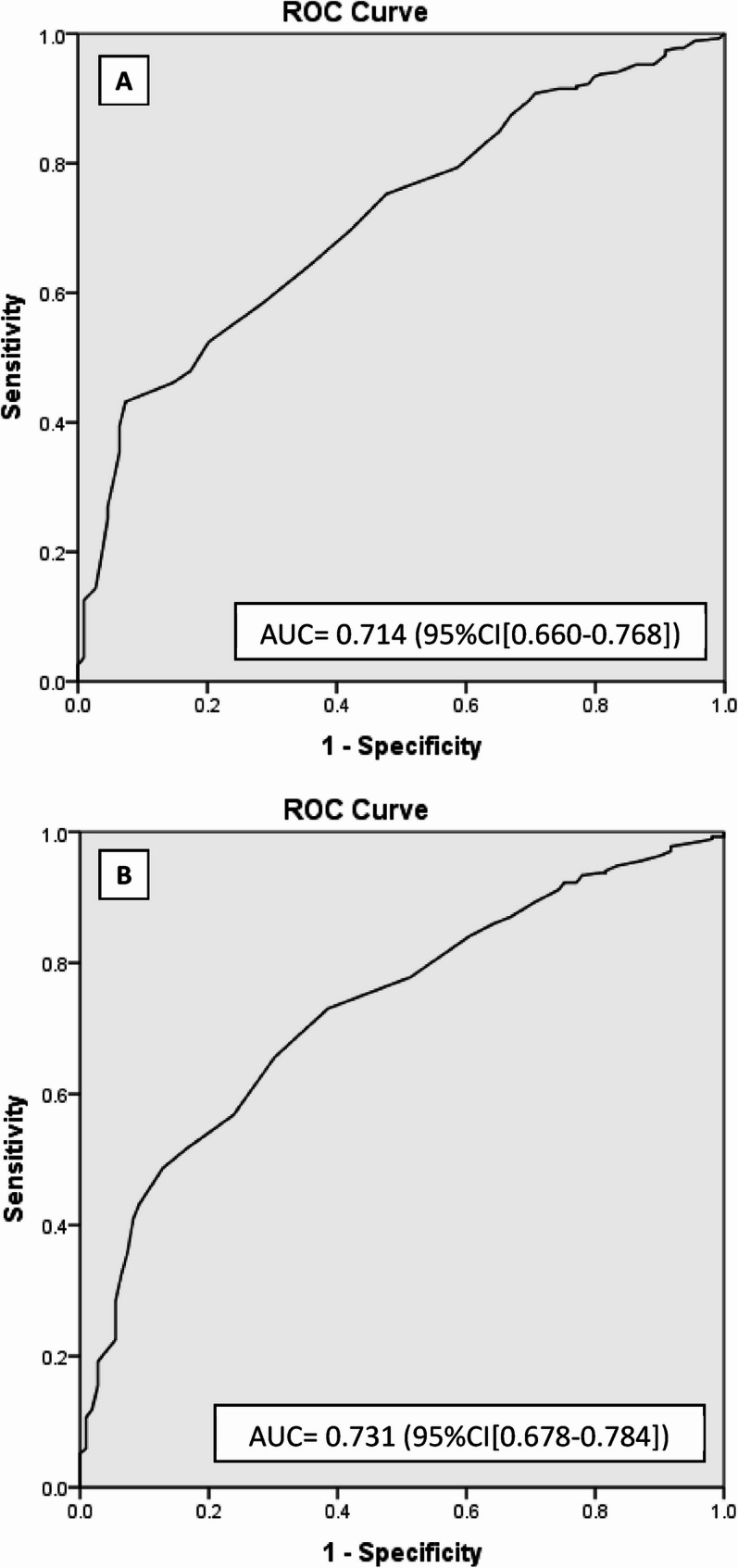
Area under ROC curve (AUC) of the 27-item (**A**) and 20-item (**B**) scales for identifying women who did not vaccinated during pregnancy

The area under the ROC curve (AUC) for the 27-item and 20-item scales was 0.714 (95% CI: 0.660–0.768) and 0.731 (95% CI: 0.678–0.784), respectively. These values indicate a moderate ability of both scales to differentiate between vaccine-hesitant and non-hesitant pregnant women, with the 20-item scale showing a slightly improved discriminative power (Fig. 3). Different cut-off scores of the 20-item scale were assessed according to the coordinates of a ROC curve (Box S2; Supplementary Material). A cut-off score of 64 on the 20-item scale was selected as it provided a sensitivity of 89.3% and a specificity of 29.4%, offering a balance between identifying vaccine-hesitant individuals and minimizing false positives (see Table 3 for cut-off analysis). Lower cut-offs, such as 60, increased specificity but at the cost of reduced sensitivity.

**Table 3:** Performance of a 20-item questionnaire in detecting those who were not vaccinated during pregnancy (*N* = 380*)

Cut-off	Sensitivity	Specificity	PPV	NPV	Accuracy
< 60	77.9%	48.6%	79.0%	46.9%	69.5%
< 64	89.3%	29.4%	75.9%	52.5%	72.1%
< 70	93.7%	18.3%	74.1%	54.1%	72.1%
< 74	96.3%	10.1%	72.7%	52.4%	71.5%

*PPV* Positive predictive value, *NPV* Negative predictive value

*Women with complete COVID-19 vaccination before pregnancy (*n* = 22) were excluded from the analyses

## Discussion

### Development and validation of the 20-item survey

The shortened 20-item survey demonstrated comparable psychometric performance to the original 27-item version, suggesting that it is a valid and reliable tool for assessing COVID-19 vaccine attitudes among pregnant women. Importantly, the reduced length may facilitate its use in clinical settings and large-scale surveys, reducing respondent burden without compromising data quality. The practical outcomes of our study include the development of a reliable tool to assess the causes of vaccine hesitancy among pregnant women. A key advantage of using a standardized instrument is that it enables comparability of factors across different populations and settings, thereby enhancing the study of vaccine hesitancy in this group.

The original instrument consisted of 27 questions based on the SAGE 5 C’ model, Behavior and Social Drivers of Vaccination (BeSD), the Health Belief Model (HBM), and the Socioecological Model and addressing attitudes and knowledge about COVID-19 disease and vaccines [16, 26–28]; both 27- and 20-item questionnaires showed a very high reliability. Nevertheless, seven questions could be removed without compromising its psychometric properties and performance in our study population. The questions removed from the 27-item survey seemed, therefore, redundant to the other questions that already properly covered the relevant topics. The high Cronbach’s alpha values (0.912 for the 27-item scale and 0.921 for the 20-item scale) indicate strong internal consistency, suggesting that the items within each scale are closely related and measure the same underlying construct. The slight increase in alpha from 0.912 to 0.921 after removing redundant items reflects an improvement in the scale’s reliability. This is particularly meaningful because it demonstrates that the shorter version maintains its reliability (validity) despite having fewer items. A meta-analysis of 20 studies noted that the number of participants’ responses was greater when the questionnaire was shorter (*p* < 0.0011), although the response varied depending on the content of the instrument [34]. Therefore, a shorter questionnaire tends to be better for application. A shorter, yet equally reliable instrument might be more easily integrated into routine clinical practice, enabling quicker and more practical assessment of vaccine acceptance [35–37].

The five factors comprising our 20-item instrument are theoretically grounded in the frameworks that informed the development of the original instrument. The multidimensional nature of the assessment provides actionable insights for addressing vaccine hesitancy. They covered (1) perceived benefits and safety; (2) concerns and misconceptions; (3) social and community influences; (4) trust in information sources; and (5) trust in government and community recommendations. By addressing these dimensions, the assessment captures the individual, interpersonal, and societal factors that contribute to vaccine hesitancy, making it highly representative of the complex decision-making process.

### Comparison with other vaccine hesitancy instruments

Other studies have already used scale-based instruments to assess vaccine acceptance and hesitancy. A cross-sectional study conducted in Serbia collected data from a national sample of adult citizens to assess vaccine acceptance [38]. They used an instrument based on the 3 C model, named the COVID-19 Vaccine Hesitancy Questionnaire (COVID-19 VHQ), with eight questions using a 5-point Likert scale. It was not clear whether a neutral category was included. The level of consistency was considered good, with a Cronbach’s alpha of 0.87 [38]. A study carried out in Hungary with 1,503 adults developed a validated instrument to identify vaccine hesitancy in the general population, named the Multidimensional Covid-19 Vaccine Hesitancy Scale (CoVaH), composed of 15 items using a 5-point Likert-scale, subdivided into skepticism, risk perception and fear of the COVID-19 vaccine [39]. This scale demonstrated a very good fit (KMO = 0.94) and internal consistencies (α values >0.89), and it was determined to possess adequate convergent, concurrent and discriminant validity in identifying COVID-19 vaccine hesitancy in the general population [39]. An online survey of 5,114 adults from the UK developed a 7-item instrument called the Oxford COVID-19 vaccine hesitancy scale, coded with a five-point Likert scale, plus on “I don`t know” option, which was excluded from scoring, identifying four factors through factor analysis [40].

A study conducted by Cheng et al. developed and validated a Public Vaccination Attitudes Scale tailored for public health crises to address vaccine hesitancy [41]. The scale was created during the COVID-19 pandemic through literature review, expert consultations and incorporated both quantitative and qualitative data. Its psychometric properties were evaluated using exploratory and confirmatory factor analyses with data from almost 4,000 respondents (general population). The final 17-item scale showed strong reliability (Cronbach’s α = 0.874) and valid constructs, with factors explaining 68.044% of the variance. The final version consisted of four factors: recognition, environment, value, and safety. The scale included both extrinsic and intrinsic factors centered on vaccine acceptance. In contrast to the scales above, our instrument did not use a neutral opinion due to the difficulty in analyzing whether persons with neutral opinions would accept a COVID-19 vaccine.

### In general, advantages and limitations of likert scales

Although it is common to use scale-based instruments to measure vaccine acceptance and hesitancy, there are few studies focusing on pregnant women and context-specific considerations of the population. The concerns of pregnant women differ from those of the general population due to the fear of complications during pregnancy and fetal development, which can increase vaccine hesitancy in this group [4, 5, 42–44].

While a dichotomous scale can be used in an instrument such as this, the Likert-scale has greater sensitivity, validity and reliability [45]. This type of scale is extensively used in epidemiological research [46]. However, it is important to note that Likert scales can lose significant information, as they are discrete categorical metrics and have the potential to introduce researcher biases into the statistical analysis [46].

### Context-specific considerations and adaptation to Brazil

A scoping review of COVID-19 vaccine hesitancy in Latin America and Africa showed that a minority of the studies (6/94) used instruments based on WHO SAGE publications [47]. There is a lack of instruments regarding vaccine acceptance and hesitancy in the Brazilian context, which differs from other countries. Although Brazil has a national immunization program which offers several vaccines free of charge, in recent years, there has been an increase in movements against vaccination and growing doubts about its safety and effectiveness, with the return of diseases previously eradicated by vaccination, such as measles [48, 49]. This manuscript describes the testing and adaptation of a survey regarding COVID-19 vaccination in pregnancy to the Brazilian context.

### Scoring and performance analysis

Our 20-item instrument delivered a score ranging from 20 to 80, depending upon the responses of the pregnant women. We assessed different cut-offs based on the coordinates of the ROC curve; however, we do not intend to propose a unique cut-off. The use of cut-offs could inform performance, anticipating the expected sensitivity of the instrument. Considering our 20-item scale, a cut-off scores close to 64 would result in balanced sensitivity and specificity, as well as higher accuracy. Scales with cut-off points can be an important tool, allowing classification of participants according to risk categories [50].

### Generalizability and future applications

While the 20-item instrument shows promise for assessing vaccine attitudes among pregnant women in Southeast Brazil, its generalizability to other regions with different cultural, economic, and healthcare landscapes requires further study. For instance, attitudes towards vaccination may vary significantly between populations with varying levels of access to healthcare services [51] Therefore, validation studies in other parts of Brazil are crucial for ensuring the broader applicability of this tool; this instrument could be applied to understand knowledge, attitudes, and practices (KAP) for other vaccines, although it would require appropriate adaptations.

The development and implementation of psychometric scales can be instrumental in assessing vaccination attitudes during public health crises, informing prioritization of vaccine policies [41, 52]. A user-centered approach to developing tools for measuring behavioral and social drivers of vaccination emphasizes the importance of understanding local contexts [53]. This approach ensures that interventions are culturally and contextually appropriate, enhancing their effectiveness. Understanding social cognition models to predict vaccine uptake is possible by identifying predictors such as instrumental attitudes and subjective norms. From this understanding, healthcare settings can design interventions that enhance vaccine acceptance, particularly among non-early adopters [54].

### Limitations and strengths

This study has several limitations. The number of women who refused to participate was significant. Non-response bias can significantly impact the findings of cross-sectional studies on vaccination decision-making [55–57]. Molinari et al. quantified bias in a health survey and found that non-response was a significant contributor to total survey error, although the overall error was small. They highlighted the importance of adjusting survey weights to correct for this bias [56]. We were unable to assess the reasons for their refusal or the characteristics of non-respondents, which limits our ability to understand the potential impact of refusal bias. If certain groups are underrepresented or excluded from the study due to non-response, the findings may not reflect the broader population. Considering the subject of vaccination and the recruitment of participants from healthcare facilities, social desirability bias is somewhat expected. The face-to-face administration of the survey may have introduced social desirability bias, as participants might have provided responses they perceived as more acceptable. Additionally, the absence of a neutral option on the Likert scale could have forced respondents to choose between positive and negative responses when they did not have any opinion, potentially skewing results. Furthermore, the sample was drawn from two hospitals in Southeast Brazil, which may limit the generalizability of our findings to pregnant women in other regions of the country with different socio-economic or healthcare access profiles. We employed some strategies to minimize this potential bias, including: assuring respondents that their answers will remain anonymous and confidential, which may encourage honesty, particularly for sensitive topics; framing some of the questions around group behaviors or societal trends rather than personal behaviors, which can decrease the pressure to respond in a socially desirable manner; conducting a pilot evaluation by experts to identify potential questions that may lead to social desirability bias, allowing for adjustments before the main survey. We would like to acknowledge that our choice to select cut-off points close to the balanced performance of the instrument, after examining the coordinates of the resulting curve, was arbitrary; the choice was not based on clinical or practical significance. However, they can assist in interpreting the performance metrics and highlight the potential clinical implications of various cut-off levels. By emphasizing these specific points on the ROC curve, it becomes evident that beyond a certain threshold, there is no significant increase in accuracy: the performance metrics plateau. This approach facilitates a comprehensive understanding of the balance between sensitivity and specificity at these cut-off levels, underscoring the inherent trade-offs involved in diagnostic decision-making. Finally, the definition of “incomplete vaccination” to determine vaccine hesitancy in pregnancy, based on Brazilian recommendations, may be quite stringent compared with other settings. However, because SAGE globally recommends a dose during each pregnancy, this analysis likely reflects a comparison of those who were and were not vaccinated in pregnancy.

Our work also has a notable strength. This approach appears to be novel, as we were unable to find other studies evaluating the psychometric properties of an instrument designed to measure knowledge, attitudes, and practices regarding COVID-19 vaccination in the obstetric population.

## Conclusion

The 20-item instrument demonstrated strong reliability and internal consistency, making it a valuable tool for assessing COVID-19 vaccine attitudes and knowledge among pregnant women in Brazil. Its concise format facilitates easier administration without compromising accuracy. With further validation, it could help inform vaccination strategies and support maternal and neonatal health during pandemics and beyond.

## Supplementary Information

Supplementary Material 1.

## Data Availability

The data that support the findings of this study are available from the research group but restrictions apply to the availability of these data, which were used under license for the current study, and so are not publicly available. Data are however available from the authors upon reasonable request and with permission of the research group and all ethical review board and institutions involved.
