# Supplementary material for: Properties and performance of a questionnaire assessing COVID-19 vaccine knowledge and attitudes in Brazilian pregnant women
Source: BMC Public Health. 2025 Nov 24;25:4121. doi: 10.1186/s12889-025-25102-z (PMC12642217; doi:10.1186/s12889-025-25102-z)
Supplement: Supplementary file 1 — Supplementary Material 1. [file 12889_2025_25102_MOESM1_ESM.docx]

**Supplementary Material**

**Table S1.** Characteristics of the study population (N=402)

|  | **N (%)** |
| --- | --- |
| **Age (Years)** |  |
| <18/emancipated | 6 (1.5) |
| 18-24 | 109 (27.1) |
| 25-34 | 198 (49.3) |
| >34 | 89 (22.1) |
| **Ethnicity** |  |
| White | 169 (42.0) |
| Pardo/black | 229 (57.0) |
| Other | 4 (1.0) |
| **Education** |  |
| Primary | 96 (23.9) |
| Secondary | 257 (63.9) |
| College or more | 49 (12.2) |
| **Without Partner** | 139 (34.5) |
| **No children<18** | 112 (41.3) |
| **Gestational age at inclusion** |  |
| 1st trimester (≤14 weeks) | 133 (33.1) |
| 2nd trimester (15-27 weeks) | 135 (33.6) |
| 3rd trimester (≥ 28 weeks) | 134 (33.3) |
| **COVID-19 Vaccinated** | 388 (96.5) |
| **Vaccination status** |  |
| Vaccinated during pregnancy | 109 (27.1) |
| Not vaccinated during pregnancy and incomplete vaccination | 257 (63.9) |
| Not vaccinated during pregnancy and full vaccination | 22 (5.5) |
| Not vaccinated | 14 (3.5) |
| **Number of COVID-19 vaccine doses*** |  |
| 1-2 | 116 (29.9) |
| 3 | 151 (38.9) |
| 4-5 | 121 (31.2) |

*Only those who were vaccinated were considered PHCU: Primary Health Care Unit; HRCU: High Risk Care Unit

**Box S1.** List of questions of the 27-item questionnaire

| Code | Description of the question | Included in the 20-item version |
| --- | --- | --- |
| Q1 | I worry that I could get COVID-19 while I am pregnant. |  |
| Q2 | COVID-19 is dangerous for pregnant people. |  |
| Q3 | COVID-19 is more dangerous for pregnant people than for people who are not pregnant. |  |
| Q4 | Getting the COVID-19 vaccine will reduce my risk of getting COVID-19 during my pregnancy. | X |
| Q5 | Getting the COVID-19 vaccine while I am pregnant will reduce my baby’s risk of getting COVID-19. | X |
| Q6 | I am confident that getting the COVID-19 vaccine during my pregnancy is safe for me. | X |
| Q7 | I am confident that getting the COVID-19 vaccine during my pregnancy is safe for my baby. | X |
| Q8* | I worry that the ingredients in the COVID-19 vaccine given to me during pregnancy are not safe for me. | X |
| Q9* | I worry that the ingredients in the COVID-19 vaccine given to me during pregnancy are not safe for my baby. | X |
| Q10* | I worry that the ingredients in the COVID-19 vaccine may negatively impact my fertility. | X |
| Q11* | I do not want to put the COVID-19 vaccine into my body when I am pregnant because I think it is unnatural. | X |
| Q12* | Vaccines improve your body’s ability to fight off diseases; this is known as immunity. I believe it is better for my body to develop immunity by getting sick than by getting the COVID-19 vaccine. | X |
| Q13 | I am in control of the decision to get or not get a vaccine during my pregnancy. |  |
| Q14 | I am in control of whether or not I can get vaccines during my pregnancy. | X |
| Q15 | If I wanted to get the COVID-19 vaccine or a booster dose, and it was available in my community for pregnant people, I am confident I could get the vaccine. | X |
| Q16 | The majority of my friends would encourage me to get the COVID-19 vaccine during my pregnancy. | X |
| Q17 | My family holds considerable influence on my decision to get the COVID-19 vaccine. |  |
| Q18 | My family would encourage me to get the COVID-19 vaccine during my pregnancy. | X |
| Q19 | The majority of my pregnant friends and family would get or have gotten the COVID-19 vaccine while they are pregnant. | X |
| Q20 | I have/had most of the important information I need/needed to make a decision about the COVID-19 vaccine during pregnancy. | X |
| Q21 | I know enough about the safety of the COVID-19 vaccine to make/have made a decision about getting the vaccine for myself while pregnant. | X |
| Q22 | I trust the information that I have received from my health care provider about use of the COVID-19 vaccine during pregnancy. | X |
| Q23 | I trust the information provided by scientists about vaccines during pregnancy. | X |
| Q24 | I trust the information that I have heard from the media about use of the COVID-19 vaccine during pregnancy. |  |
| Q25 | The government recommends pregnant people receive the COVID-19 vaccine while pregnant. | X |
| Q26 | I trust my government and politicians’ recommendations on COVID-19 vaccination for pregnant women. | X |
| Q27 | Leaders in my community recommend that pregnant people receive the COVID-19 vaccine while pregnant. | X |

*In the scale, inverted scores were considered for questions in red (Q8-Q12).

**Table S2.** Pearson’s correlation coefficient between question of the question 20-item questionnaire

|  | Q4 | Q5 | Q6 | Q7 | Q8 | Q9 | Q10 | Q11 | Q12 | Q15 | Q16 | Q18 | Q19 | Q20 | Q21 | Q22 | Q23 | Q25 | Q26 | Q27 |
| --- | --- | --- | --- | --- | --- | --- | --- | --- | --- | --- | --- | --- | --- | --- | --- | --- | --- | --- | --- | --- |
| Q4 | 1.000 | .554 | .628 | .383 | .361 | .235 | .444 | .356 | .370 | .368 | .398 | .390 | .279 | .321 | .335 | .304 | .231 | .389 | .302 | .386 |
| Q5 | .554 | 1.000 | .790 | .551 | .551 | .405 | .636 | .457 | .470 | .347 | .495 | .440 | .329 | .353 | .402 | .441 | .301 | .506 | .435 | .422 |
| Q6 | .628 | .790 | 1.000 | .516 | .528 | .349 | .582 | .480 | .441 | .369 | .499 | .438 | .395 | .384 | .402 | .425 | .319 | .466 | .440 | .435 |
| Q7 | .383 | .551 | .516 | 1.000 | .736 | .389 | .506 | .341 | .441 | .264 | .334 | .245 | .206 | .299 | .386 | .398 | .240 | .318 | .417 | .223 |
| Q8 | .361 | .551 | .528 | .736 | 1.000 | .404 | .516 | .294 | .386 | .283 | .368 | .308 | .273 | .344 | .357 | .391 | .275 | .312 | .365 | .221 |
| Q9 | .235 | .405 | .349 | .389 | .404 | 1.000 | .510 | .423 | .343 | .093 | .234 | .189 | .220 | .231 | .302 | .196 | .040 | .298 | .231 | .234 |
| Q10 | .444 | .636 | .582 | .506 | .516 | .510 | 1.000 | .529 | .472 | .213 | .475 | .347 | .302 | .309 | .428 | .350 | .216 | .451 | .451 | .341 |
| Q11 | .356 | .457 | .480 | .341 | .294 | .423 | .529 | 1.000 | .406 | .186 | .341 | .234 | .220 | .174 | .343 | .307 | .154 | .327 | .216 | .339 |
| Q12 | .370 | .470 | .441 | .441 | .386 | .343 | .472 | .406 | 1.000 | .153 | .405 | .266 | .299 | .296 | .541 | .433 | .203 | .393 | .330 | .366 |
| Q15 | .368 | .347 | .369 | .264 | .283 | .093 | .213 | .186 | .153 | 1.000 | .447 | .485 | .317 | .259 | .239 | .286 | .245 | .260 | .280 | .257 |
| Q16 | .398 | .495 | .499 | .334 | .368 | .234 | .475 | .341 | .405 | .447 | 1.000 | .458 | .247 | .294 | .412 | .443 | .244 | .477 | .373 | .419 |
| Q18 | .390 | .440 | .438 | .245 | .308 | .189 | .347 | .234 | .266 | .485 | .458 | 1.000 | .490 | .344 | .302 | .298 | .188 | .427 | .185 | .421 |
| Q19 | .279 | .329 | .395 | .206 | .273 | .220 | .302 | .220 | .299 | .317 | .247 | .490 | 1.000 | .564 | .480 | .342 | .166 | .339 | .227 | .342 |
| Q20 | .321 | .353 | .384 | .299 | .344 | .231 | .309 | .174 | .296 | .259 | .294 | .344 | .564 | 1.000 | .454 | .467 | .297 | .286 | .366 | .348 |
| Q21 | .335 | .402 | .402 | .386 | .357 | .302 | .428 | .343 | .541 | .239 | .412 | .302 | .480 | .454 | 1.000 | .460 | .202 | .379 | .346 | .402 |
| Q22 | .304 | .441 | .425 | .398 | .391 | .196 | .350 | .307 | .433 | .286 | .443 | .298 | .342 | .467 | .460 | 1.000 | .371 | .351 | .479 | .399 |
| Q23 | .231 | .301 | .319 | .240 | .275 | .040 | .216 | .154 | .203 | .245 | .244 | .188 | .166 | .297 | .202 | .371 | 1.000 | .249 | .514 | .176 |
| Q25 | .389 | .506 | .466 | .318 | .312 | .298 | .451 | .327 | .393 | .260 | .477 | .427 | .339 | .286 | .379 | .351 | .249 | 1.000 | .367 | .598 |
| Q26 | .302 | .435 | .440 | .417 | .365 | .231 | .451 | .216 | .330 | .280 | .373 | .185 | .227 | .366 | .346 | .479 | .514 | .367 | 1.000 | .328 |
| Q27 | .386 | .422 | .435 | .223 | .221 | .234 | .341 | .339 | .366 | .257 | .419 | .421 | .342 | .348 | .402 | .399 | .176 | .598 | .328 | 1.000 |

**
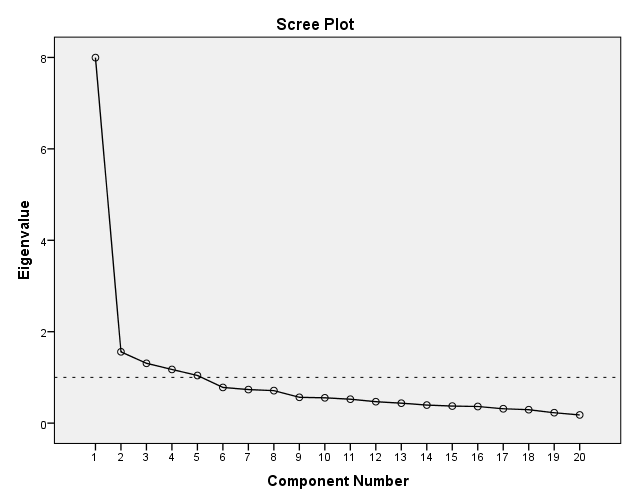
Figure S1**. Eigenvalues plotted against the corresponding principal components

**Box S2.** Coordinates of the ROC curve of the 20-item scale

| **Positive if Less Than or Equal To** | **Sensitivity** | **1 - Specificity** | **Positive if Less Than or Equal To** | **Sensitivity** | **1 - Specificity** |
| --- | --- | --- | --- | --- | --- |
| 30.0000 | .000 | .000 | 58.5000 | .731 | .385 |
| 32.0000 | .004 | .000 | 59.5000 | .779 | .514 |
| 35.0000 | .007 | .000 | 60.5000 | .841 | .606 |
| 37.5000 | .015 | .000 | 61.5000 | .860 | .642 |
| 38.5000 | .026 | .000 | 62.5000 | .871 | .670 |
| 40.0000 | .033 | .000 | 63.5000 | .893 | .706 |
| 41.5000 | .052 | .000 | 64.5000 | .911 | .743 |
| 42.5000 | .059 | .009 | 65.5000 | .923 | .752 |
| 43.5000 | .089 | .009 | 66.5000 | .923 | .771 |
| 44.5000 | .107 | .009 | 67.5000 | .934 | .780 |
| 45.5000 | .118 | .018 | 68.5000 | .937 | .807 |
| 46.5000 | .155 | .028 | 69.5000 | .937 | .817 |
| 47.5000 | .192 | .028 | 70.5000 | .941 | .817 |
| 48.5000 | .225 | .055 | 71.5000 | .948 | .835 |
| 49.5000 | .284 | .055 | 72.5000 | .956 | .872 |
| 50.5000 | .325 | .064 | 73.5000 | .963 | .899 |
| 51.5000 | .358 | .073 | 74.5000 | .970 | .917 |
| 52.5000 | .410 | .083 | 75.5000 | .978 | .917 |
| 53.5000 | .432 | .092 | 76.5000 | .985 | .963 |
| 54.5000 | .487 | .128 | 77.5000 | .989 | .982 |
| 55.5000 | .517 | .165 | 78.5000 | .993 | .982 |
| 56.5000 | .568 | .239 | 79.5000 | .993 | 1.000 |
| 57.5000 | .657 | .303 | 81.0000 | 1.000 | 1.000 |
